# Behaviour Change Techniques and Mechanisms of Action: Identification of the Active Ingredients in Communication Partner Training for People With Acquired Brain Injury

**DOI:** 10.1111/1460-6984.70238

**Published:** 2026-04-13

**Authors:** Nicholas Behn, Madeline Cruice, Katerina Hilari, Leanne Togher, Fiona Johnson, Ian Kellar

**Affiliations:** ^1^ School of Health and Medical Sciences City St George's, University of London London UK; ^2^ Faculty of Medicine and Health The University of Sydney Sydney Australia; ^3^ Linguistic Resolutions London UK; ^4^ Department of Psychology The University of Sheffield Sheffield UK

**Keywords:** behaviour change, brain injury, cognitive‐communication, rehabilitation, social communication, communication partner training

## Abstract

**Background:**

Cognitive‐communication disorders (CCD) are common after acquired brain injury (ABI) and can have a negative impact on a person's life. Training a communication partner can improve the communication skills of the person with ABI; however, families are dissatisfied with existing communication partner training (CPT) and support. There is emerging evidence for the effectiveness of training for communication partners (CPs), though there is variability in the content, dosage and delivery of training; and published programmes are rarely used by speech and language therapists in practice. The strongest evidence is for a single programme, TBI Express, which has three different versions. Therefore, the aim of this study is to identify and describe the active components of existing training programmes for people with CCD.

**Method:**

The treatment manuals from each of the training programmes (i.e., TBI Express, TBI Express‐Adapted, and TBIconneCT) were coded using the BCTTv1. Mechanisms of action (MoAs) and each behaviour change technique (BCT) was identified and linked using the Theory and Techniques Tool. Prior to coding, pilot coding was completed on two modules from TBI Express with reliability of coding established (kappa and % agreement) for two modules of the remaining two programmes representing ∼20–33% of treatment content. Coding disagreements were recorded and described and subsequently resolved.

**Results:**

Across the three programmes, between 20 and 25 BCTs were identified and comprised 27 unique BCTs across all manuals. These BCTs were linked to between 15–16 identified MoAs and comprised 16 unique MoAs. The most common BCTs were providing *information about social and environmental consequences*; *instruction on how to perform the behaviour*; *behavioural practice/rehearsal*; and *feedback on the behaviour*. The most common mechanisms targeted were *beliefs about capabilities; skills; and behavioural regulation*. Reliability of coding BCTs and MOAs was moderate‐to‐almost‐perfect (kappa = 0.69–0.88, 73–85% agreement). Disagreements in coding were discussed and resolved through consensus agreement.

**Conclusions:**

Applying behaviour change theory to TBI Express has revealed unique insight into the active ingredients of training. CPs are anticipated to improve their communication behaviours via capabilities, skills and regulation, through SLT‐delivered CPT which focuses most commonly on information provision, instruction, rehearsal, and feedback. Such insights are gathered to further refine and adapt existing interventions. Further work is needed to identify the most important active ingredients to expert clinicians, to design and test the feasibility of an adapted CPT programme for implementation in public healthcare services.

**WHAT THIS PAPER ADDS:**

*What is already known on this subject*

- Training a communication partner can improve the communication skills of people with ABI however; families are dissatisfied with existing education, training and support. Speech and language therapists face barriers in the workplace and there is variability in the range of evidence‐based programmes for training communication partners.

*What this study adds to existing knowledge*

- Through exploration of three existing evidence‐based communication partner training programmes, this study identifies the active components required to deliver training to individuals with ABI and their communication partners. Mechanisms of change and behaviour change techniques are identified for each of the three programmes, highlighting areas of commonality between the three programmes.

*What are the potential or actual clinical implications of this study?*

- Training communication partners is a complex and multicomponential intervention. Communication partners are anticipated to improve their communication behaviours via capabilities, skills and regulation, through training that focuses on information provision, instruction, rehearsal, and feedback. This research gives therapists an understanding of what is important when training communication partners and provides insight to researchers to refine and adapt existing training programmes.

## 1 Introduction

Impaired communication is a long‐term and pervasive challenge for people with acquired brain injury (ABI: Knox and Douglas 2009; Olver et al. 1996; Ponsford et al. 2014; Snow et al. 1998). The economic burden of brain injury is substantial, with costs reaching an estimated £15 billion each year in the UK (Centre for Mental Health 2016) and £282 billion worldwide (Maas et al. 2017). Over 1.3 million people live in the UK with the consequences of brain injury, with nearly 350,000 hospital admissions per year (Centre for Mental Health 2016; Headway 2015). More than two‐thirds of individuals who sustain an ABI whether traumatic (e.g. from road traffic accidents, falls) or non‐traumatic (e.g. tumours, encephalitis, anoxic damage) experience some form of cognitive communication disorder (CCD: Hewetson et al. 2017; Kelly et al. 2017; Shorland et al. 2022).

The communication impairments individuals experience post‐injury are diverse. These include excessive or reduced speech output, perseveration, tangential speech, difficulties with initiation and/or turn taking, poor listening, disruptive behaviours, and socially inappropriate interactions (Coelho et al. 1991; Hartley and Jensen 1992; Sim et al. 2013; Spence et al. 1993). These impairments significantly hinder a person's relationships with others (Grayson et al. 2021; Kelly et al. 2023), return to work (Meulenbroek and Turkstra 2016; Rietdijk et al. 2013), independence, social integration (Dahlberg et al. 2006; Knox and Douglas 2009; Struchen et al. 2008) and overall quality of life (Dahlberg et al. 2006; Galski et al. 1998).

Given the complexity and heterogeneity of these challenges (Hartley and Jensen 1992; Snow et al. 1997), interventions must address both the communication skills of individuals with ABI and the broader environment in which communication occurs (Ylvisaker et al. 1993). Communication partners (CPs), including family members, friends, or caregivers, play a crucial role in shaping communication environments and fostering positive psychosocial outcomes (Foster et al. 2012; Macaden et al. 2010; Stergiou‐Kita et al. 2011). However, supporting individuals with ABI can also be a source of emotional and psychological distress for CPs, impacting family functioning and well‐being (Anderson et al. 2002; Grayson et al. 2020; Vangel et al. 2011). These challenges can persist for years post‐injury (Grayson et al. 2020). Importantly, the communication strategies employed by CPs can enhance or hinder interactions (Shelton and Shryock 2007; Togher et al. 1997). For example, frequent testing of knowledge, limiting participation opportunities and/or not giving the individual with ABI a chance to respond can all hinder communication (Mann et al. 2015; Sim et al. 2013). Conversely, using a supportive questioning style and positive communication strategies (e.g., use of short, simple direct sentences and questions) can facilitate better interactions (Mann et al. 2015; Shelton and Shryock 2007). Despite these insights, more than 60% of families report dissatisfaction with existing training and support services in relation to CCD and feel their rehabilitation needs for CCD are not met (Grayson et al. 2020). Families want information on patterns of CCD recovery and the long‐term impact of CCD changes (Grayson et al. 2020). However, the time to create accessible information and tailor the format and content to the individual's CCD changes can be a barrier, as can a person's readiness to receive and engage with the information (Short et al. 2014). This unmet need reported by families has persisted for almost two decades from the early months to many years post‐injury (Bond et al. 2003; Dillahunt‐Aspillage et al. 2013; Grayson et al. 2020; Nielsen et al. 2019).

Communication partner training (CPT) has demonstrated efficacy in improving communication skills and psychosocial outcomes for people with ABI and their CPs (Behn et al. 2021; Wiseman‐Hakes et al. 2020). While speech and language therapy for individuals with ABI can improve their communication skills (Finch et al. 2016), research indicates that CPT involving *both* the individual with ABI and CP is superior (Togher et al. 2013). International guidelines (Togher et al. 2023) and systematic reviews (Behn et al. 2021; Wiseman‐Hakes et al. 2020) endorse CPT as a best practice intervention. Clinical guidelines and/or practice standards for SLTs working with people with ABI in the UK (British Society of Rehabilitation Medicine 2009; RCSLT 2010; Scottish Intercollegiate Guidelines Network 2013) and internationally (American Speech Language and Hearing Association n.d.; Bayley et al. 2016; CALSPO 2015; Togher et al. 2014) advocate for CPT. Training CPs can help to foster effective and supportive communication, strengthen relationships, enhance social participation, and reduce psychological burden (Togher et al. 2016).

A systematic review and meta‐analysis demonstrated the effectiveness of CPT programmes from three randomised controlled trials, two non‐randomised controlled trials and three single‐case experimental designs. A meta‐analysis of two controlled trials (both of ‘good’ methodological quality) revealed moderate‐to‐large effects for improving the skills of individuals with ABI (*n* = 36; SMD 0.31–0.50) and their CPs (*n* = 41; SMD 0.97–1.05) (Behn et al. 2021). Improved communication skills facilitate stronger relationships, greater social participation, increased confidence and enhanced independence (Togher et al. 2012a). However, despite promising results, significant variability exists in CPT programme content, dosage and delivery (Behn et al. 2021; Wiseman‐Hakes et al. 2020). Program duration ranges from a few minutes to 48 h; with diverse delivery methods including face‐to‐face, telehealth, DVDs, written information, and individual or group sessions. The strongest evidence is drawn from a non‐randomised controlled trial of ‘good’ quality and supports the manualised TBI Express programme, a 35‐h/10‐week intervention delivered in‐person in both group and individual sessions to people with brain injury and their partners (Togher et al. 2013). Adaptations such as TBI Express‐Adapted, a 17‐h/8‐week intervention delivered in group sessions to partners only; and TBI ConneCT, a 15‐h/10‐week intervention delivered online to a person with brain injury and their partner, have shown comparable efficacy in randomised controlled trials with lower intensity and alternative delivery methods (Behn et al. 2012; Rietdijk et al. 2020). Given the variability in dosage and intensity, optimising interventions for implementation in clinical settings is a key research priority (Maas et al. 2017).

Despite CPT being a recommended intervention, its implementation in clinical practice varies. Speech and Language Therapists (SLTs) specialise in working with communication impairments after ABI, and help CPs develop the skills they need to support and facilitate better communication skills in the individual with ABI. A UK survey of 169 SLTs found that nearly 60% reported they did not provide training consistent with best practice, with 70% of inpatient rehabilitation SLTs reporting that they were unable to deliver CPT at the recommended intensity (Behn et al. 2020). SLTs were motivated to provide CPT but faced barriers such as limited time, resources, staffing, clinical skills, and workplace support (Behn et al. 2020). Notably, TBI Express was used by only 10–20% of therapists, often as a rough guide rather than a structured intervention. The program's duration (10 weeks) and intensity (3.5 h/week) is likely to have made implementation challenging within public sector settings.

To improve the efficiency of CPT, and ensure its adoption by SLTs, it is crucial to identify the active components of a programme (Levati et al. 2016; O'Cathain et al. 2019). By focusing on the most effective programmes (*TBI‐Express*, and its variants) these components can be extracted and identified to help facilitate intervention refinement for broader implementation.

Active components may be described in terms of Behaviour Change Techniques (BCTs) and Mechanisms of Action (MoAs). Established frameworks such as the BCT taxonomy (BCTTv1) (Michie et al. 2013) have been used to analyse the presence of BCTs which refer to the *core techniques or ingredients* used by a therapist to *bring about a change in behaviour* in a person. This taxonomy describes 93 BCTs across 19 groups (e.g., goals and planning, feedback and monitoring, social support). These techniques can be identified from published papers (Howlett et al. 2019) or intervention manuals (Lorencatto et al. 2013), with more BCTs shown to be identified from manuals than published studies alone. CPT manuals in stroke and aphasia have previously been used to identify the presence of BCTs (Johnson et al. 2021). However, many of these studies have not identified the linked MoA which refer to the *process* through which the BCTs are likely to have *influenced change* in behaviour (Carey et al. 2019). Therefore, it is proposed by identifying the BCTs and linked MoAs, that a more optimised training programme may be created as fewer BCTs may be required, and there might be opportunity to refine the intervention to improve the deliverability in public healthcare services and reduce the burden on staff and patients.

Therefore, the aims of this study were to: (1) explore the reliability in identifying BCTs and MoAs for CPT programmes for people with CCDs after brain injury; and (2) extract the BCTs and MoAs for three existing CPT programmes (*TBI Express, TBI Express‐Adapted* and *TBIconneCT*) intended to improve the communication skills of individuals with ABI and their CPs.

## 2 Methods

### 2.1 Data

This study used three CPT manuals reported in three controlled trials (Table 1). The published versions of TBI Express (Togher et al. 2010) and TBIconneCT (Rietdijk et al. 2019) and unpublished version of TBI Express‐Adapted (Behn et al. 2012) were used for coding the BCTs and MoAs. Each manual was written for a treating clinician and provided resource materials with clear session‐by‐session instruction and activities to deliver CPT to people with ABI and their partners. Each module represents a single treatment session. Printable hand outs accompanied each session which enabled participants (*both* people with ABI and their partners) to form their own manual over the course of the training. Appendix A provides a complete list of the session modules for each of the three coded programmes.

**TABLE 1:** Description of the communication partner training programmes used for coding.

Name	TBI Express	TBI Express‐Adapted	TBIconneCT
Provider	SLT	SLT	SLT
Setting, country	Brain injury centres, Australia	Residential rehabilitation centre, UK	Online, Australia
Recipient	Person with ABI and CP	Paid CPs	Person with ABI and CP
Dosage	35 h over 10 weeks	17 h over 8 weeks	15 h over 10 weeks
Delivery method	Group and individual sessions in‐person	Group in‐person	Individual sessions via telehealth
Number of session modules^a^	10	6	10
Length of manual	226 pages^b^	178 pages	245 pages

^a^ See Appendix A for a complete list of the modules from each of the three coded programmes.

^b^ The TBI Express manual is 409 pages in length. Only sections 5, 1, 2.5 were coded as these sections are relevant to the training of communication partners; remaining sections are focused on training the person with brain injury only and not the communication partners.

### 2.2 Raters, Training and Reflexivity

The primary rater (NB) was a qualified speech and language therapist with 24 years’ experience of working with individuals with ABI. He had been involved in the development of one of the coded programmes, TBI Express‐Adapted (Behn et al. 2012; Behn et al. 2015). NB had completed formalised training in the BCTT including an online self‐led training programme that used written material, guidelines for coding and exercises to practice coding intervention descriptions (BCTTv1 online training website, www.bct‐taxonomy.com, Centre for Behaviour Change: Wood et al. 2015). The second rater (FJ) was a qualified speech and language therapist with 13 years’ experience of working with individuals with ABI. FJ was not involved in the development or evaluation of any of the coded programmes and had limited clinical experience of having delivered parts of TBI Express to individuals with ABI. She had previously completed the BCTTv1 online self‐led training programme and had experience of using the BCTTv1 to code a CPT manual, ‘Better Conversations with Aphasia’ programme (Johnson et al. 2021).

The broader research team comprised four members. Three were speech and language therapists, one of whom was involved in the development of all three coded manuals (LT). Two members of the team had experience in CPT for people with post‐stroke aphasia, and coding programmes and/or publications using the behaviour change theory, respectively (KH and MC). The final member of the team was a health psychology researcher with extensive experience of behaviour change theory, including in stroke and aphasia (IK). All authors had completed the BCTTv1 online self‐led training programme, and were involved in discussions about the coding process, challenges that arose and decisions about the final BCTs and MoAs identified from the three coded programmes.

### 2.3 Coding Procedure

The primary rater coded all 26 modules from the three CPT programme manuals. Each module contained tasks or activities, followed by hand outs and/or additional resources. The second rater rated two modules from each of the three programmes (20–33% of each programme), purposively selected by the first rater to represent a range of tasks and activities. To pilot the coding process, the raters coded two modules from the TBI Express programme first (module one on *introductions;* and module five on *elaboration*). Raters then met to discuss the codes and discrepancies and finalise a set of coding rules to guide the process for the next two programmes. In the subsequent programmes, the modules coded by both raters comprised of TBI and *communication* (module two) and *question asking* (module five) for TBI Express‐Adapted; and *playing your role* (module three) and *putting it all together* (module 10) for TBIconneCT. All coding was completed by each rater in NVivo, Version 14.

Raters examined each activity or task and made a judgement as to which BCT they corresponded to, with reference to the BCTTv1 definitions and examples. All 93 BCTs were considered for each module. The rater then recorded the number and label of the BCT next to the task or activity in NVivo. Next, the rater made a judgement as to which MoA was most likely present for each BCT using the Theory and Techniques Tool (Hall et al. 2021) to guide decision making. This online tool links a set of 26 MoAs drawn from the Theoretical Domains Framework, and frequent MoA constructs from theories of behaviour change (Carey et al. 2019; Crawshaw et al. 2023) with BCTs based on literature synthesis and expert consensus (Johnston et al. 2021). The links are shown as either conclusive, inconclusive, non‐links or no evidence; and only the conclusive or inconclusive links were considered. Raters were required to identify the 1–3 MoAs most likely to be present from the task or activity in the programme. Initially, raters coded who the BCT was directed to (person with ABI or CP) and focus of BCT (a specific goal or conversation) but this level of coding was discontinued following pilot coding as information was not consistently provided in the manuals to make these judgements.

All information was recorded in a Microsoft Excel spread sheet. There was no threshold for the number of times or number of BCTs delivered. A single instance was enough for the BCT to be coded as present as has been applied in other similar studies (Crawshaw et al. 2023; Ubhi et al. 2016; Wood et al. 2015).

Once the pilot coding for TBI Express was completed and the two raters had met to share their decisions and codes, the raters next coded the TBI Express‐Adapted manual before meeting a second time to share their findings. The raters then coded the TBIconneCT manual before meeting a third time. For each meeting, the primary rater used the coding decisions in NVivo and the Excel spread sheet to identify the discrepancies between raters to discuss at each meeting (Presseau et al. 2015). Agreement occurred when both raters coded the same BCT(s) for the same activity. Discrepancies occurred when: (1) one rater coded a BCT but the second rater did not; or (2) one rater coded a BCT(s) but the other rater coded the BCT(s) differently. Typically, a discrepancy occurred when a rater either: did not consider sufficient information was provided for a BCT to be present, or the activity description did not sufficiently match a definition of an individual BCT. Where a discrepancy occurred, the BCT and coded task or activity was discussed as were the BCT‐MoA links to resolve the discrepancies through consensus agreement. Where agreement was not reached, these discrepancies were shared with the broader research team and the outcome of any discussions recorded. The coding guidelines for BCTs and BCT‐MoA links were then clarified and updated (Carey et al. 2019), before the raters progressed to code the next programme.

To ensure credibility of the coding procedure, the first author kept a clear audit trail of the coding process with clear documentation of all discussions and decisions made between the two raters (NB and FJ) and the wider research team. The primary rater coded all remaining modules from the three programmes iteratively with re‐coding completed for all three programmes after discussions. The re‐coding of all programmes ensured there was consistency of coding following all discussions between raters and the broader research team.

### 2.4 Coding Assumptions

Several assumptions were initially made when coding. First, during the pilot coding process, it was apparent that there was not a specific underlying target behaviour (e.g., asking questions, take a turn) that was clearly defined. When this occurs, BCTs have been coded at a broader behavioural level (Presseau et al. 2015). For the purposes of coding, a change in conversation or communication was defined as the broader behavioural level. Second, each module contained session tasks and activities followed by hand outs and extra resources. The latter were often referred to as ‘provide and discuss [name of hand out]’. The degree with which the information was comprehensively discussed with participants was not clear. Here, it was agreed that the hand out would be reviewed, and it was assumed that the following two BCTs were likely to be present: *information about social and environmental consequences*, and *instruction on how to perform the behaviour*; with additional BCTs coded if judged to be present. Third, the BCTTv1 description of habit formation was unclear as the BCT was defined as repeated rehearsal of the behaviour. Therefore, if two or more instances of ‘behaviour practice/rehearsal’ were found to be present, then ‘habit formation’ was coded as present. Fourth, there was a broader discussion between raters and the team about the mechanisms of *intentions* and *motivation*; and *attitude towards the behaviour* and *general attitudes/beliefs*. Discussion revealed considerable overlap between these paired mechanisms. Therefore, for coding presence of MoAs, a more conservative approach was adopted to code to *intentions* (including motivation) and *attitude towards the behaviour* (including general attitudes/beliefs). Finally, distinguishing between behaviour and outcome was often difficult based on the information provided in the manuals. For example, the phrase ‘provide feedback about the conversation’ is ambiguous, as it is unclear whether the feedback concerns a specific behaviour or the outcome of using that behaviour. In such instances, raters agreed to be more conservative and code to the *behaviour* rather than the outcome unless explicitly specified.

### 2.5 Data Analysis

Reliability was calculated on the coded data from the TBI Express‐Adapted and TBIconneCT programmes at the level of the individual modules. Interrater reliability was assessed for the agreed presence or absence of the BCTs or MoA within each module. Reliability was assessed using Cohen's kappa and percent agreement for BCTs or MoAs present. Previous researchers have highlighted the inflation of the kappa statistic due to the agreed absence of most BCTs or a finite number of MoAs (Crawshaw et al. 2022; Gardner et al. 2016). Therefore, percentage agreement was also calculated to assess reliability (Lorencatto et al., 2013; Carey et al. 2019). Values used for interpretation of reliability were 0–0.20 and 0–4% to indicate no agreement, 0.21–0.39 and 4–15% minimal agreement, 0.40–0.59 and 15–35% weak agreement, 0.60–0.79 and 35–63% moderate agreement, 0.80–0.90 and 64–81% strong agreement and above 0.90 and 82–100% as almost perfect (McHugh 2012).

The frequency and type of identified BCTs and MoAs was quantified for each individual module across all modules within the programmes as has been done in previous studies (Presseau et al. 2015; Crawshaw et al. 2023). The BCTs and MoAs were represented using an automated heat‐map colour gradient (red to green) generated in Microsoft Excel with green indicating higher relative frequency and red indicating lower relative frequency within each programme. Colour scaling was applied independently for each programme based on its own data range

## 3 Results

### 3.1 Reliability Analyses

The level of agreement between raters for the two coded programmes is shown in Table 2. For the TBI Express‐Adapted and TBIconneCT programmes, there was strong agreement for identifying BCTs (*k* = 0.82–0.88, 75–81%) and moderate‐to‐almost perfect agreement for identifying MoAs (*k* = 0.69–0.85, 73–85%).

**TABLE 2:** Level of agreement between raters for TBI express‐adapted and TBIconneCT.

CPT programme	Agreed present/total identified	Kappa statistic	Percent agreement	Level of agreement
TBI Express—Adapted				
BCT—TBI and communication (M2)	13/16	0.88	81%	Strong
BCT—question asking (M5)	13/17	0.84	76%	Strong
MoA—TBI and communication (M2)	10/13	0.77	77%	Moderate‐to‐strong
MoA—question asking (M5)	8/11	0.79	73%	Moderate‐to‐strong
TBIconneCT				
BCT—playing your role (M3)	15/20	0.82	75%	Strong
BCT—putting it all together (M10)	15/19	0.86	79%	Strong
MoA—playing your role (M3)	10/14	0.69	79%	Moderate‐to‐strong
MoA—putting it all together (M10)	11/13	0.85	85%	Strong‐to‐almost perfect

Abbreviations: CPT, communication partner training; TBI, traumatic brain injury; BCT, behaviour change technique; MoA, mechanism of action; M2, module 2; M3, module 3; M5, module 5; M10, module 10.

Across the four modules, there were 72 BCTs identified and 16/72 (22%) discrepancies of coding (see Supplementary Material A). Half of the discrepancies (8/16, 50%) occurred as one rater had identified the presence of a BCT while the second rater had not. For several of these discrepancies, one rater had extracted information from a hand out not explicitly linked to a session activity while the second rater did not. The remaining discrepancies were either coded differently between raters (4/16, 25%), or one rater had included additional codes not identified by the second rater (4/16, 25%).

There were four discrepancies identified (4/16, 25%) for the presence of *social support* BCTs in the group. This is often related to the degree of clarity in the programmes. As a result, raters agreed to either code more conservatively to the *social support (unspecified) (3.1)* BCT or omit the BCT completely. There were also four discrepancies (4/16, 25%) related to the *goals* and *feedback* BCTs. In many cases, this related to whether the activity was coded to the behaviour or outcome. In these instances, following the setting of coding guidelines, conservative judgement meant behaviour was selected.

Across the four modules, there were 51 MoAs identified and 12/51 (24%) discrepancies of coding. Most discrepancies related to whether the mechanisms of *attitude towards the behaviour* (4/12, 33%), *emotion* (2/12, 17%), and *social influences* (2/12, 17%) were present in the modules.

### 3.2 Presence of BCTs in CPT Programmes

Across all three programmes, 27 BCTs were identified with most BCTs identified for the TBIconneCT programme (25 BCTs) and the least for TBI Express‐Adapted (20 BCTs). See Table 3. Across all three programmes, there were seven BCTs present in at least 80% of sessions. These were *goal setting (behaviour) (1.1), behavioural contract (1.8), feedback on behaviours (2.2), information about social and environmental consequences (5.3), behavioural practice/rehearsal (8.1), habit formation (8.3)* and *generalisation of target behaviour (8.6)*. A further 2 BCTs, *problem solving (1.2)* and *action planning (1.4)* were present in at least 80% of sessions for two programmes, TBI Express‐Adapted and TBIconneCT.

**TABLE 3:** Heat map and number of session modules within each programme where individual BCTs are present from most (in dark green) to least frequent (in red) and absent (black).

Behaviour change technique (BCT)	TBI Express (/10)	TBI Express‐Adapted (/6)	TBIconneCT (/10)
1.1 Goal setting (behaviour)	8	6	10
1.2 Problem solving	7	5	9
1.3 Goal setting (outcome)	2	5	3
1.4 Action planning	7	5	8
1.5 Review behaviour goal(s)	7	1	9
1.8 Behavioural contract	8	6	10
2.2 Feedback on behaviour	10	6	9
2.3 Self‐monitoring of behaviour	7	4	10
2.4 Self‐monitoring of outcome(s) of behaviour	2	1	6
2.7 Feedback on outcome(s) of behaviour	7	4	7
3.1 Social support (unspecified)	7	2	
3.2 Social support (practical)	1		1
4.1 Instruction on how to perform the behaviour	7	5	6
5.3 Information about social and environmental consequences	10	5	9
5.4 Monitoring of emotional consequences			9
6.1 Demonstration of the behaviour	5	4	1
7.1 Prompts/cues	1		4
8.1 Behavioural practice/rehearsal	10	6	10
8.2 Behaviour substitution	2	2	5
8.3 Habit formation	8	3	8
8.6 Generalisation of target behaviour	10	5	9
8.7 Graded tasks	1		
10.4 Social reward			9
12.5 Adding objects to the environment			1
13.2 Framing/reframing	3	2	4
13.4 Valued self‐identity			2
15.3 Focus on past success	2	1	8

### 3.3 Presence of MoAs in CPT Programmes

Across all three programmes, 17 MoAs were identified with most MoAs identified for the TBI Express and TBIconneCT programme (16 MoAs) and the least for TBI Express‐Adapted (14 MoAs). See Table 4. Five MoAs (*Knowledge, Skills, Beliefs about capabilities, Goals* and *Behavioural regulation*) were targeted in all sessions across the three programmes. A further four MoAs (*Beliefs about consequences, Motivation, Attitude towards the behaviour* and *Feedback processes*) were targeted in at least 80% of sessions, defined as ≥8 of 10 sessions for two programmes (i.e., TBI Express and TBIconneCT) and ≥5 of 6 sessions for the third programme (i.e., TBI Express‐Adapted). One further MoA, (*Behavioural cueing)* was present in at least 80% of sessions for two programmes (i.e., TBI Express‐Adapted and TBIconneCT).

**TABLE 4:** Number of session modules within each programme where individual MoA are present from most (in dark green) to least frequent (in red) and absent (black).

Mechanism of action (MoA)	TBI Express (/10)	TBI Express‐Adapted (/6)	TBIconneCT (/10)
Knowledge	10	6	10
Skills	10	6	10
Beliefs about capabilities	10	6	10
Beliefs about consequences	10	5	10
Reinforcement			9
Motivation^a^	10	6	9
Goals	10	6	10
Memory, attention, and decision processes	8	3	8
Environmental context and resources	1		4
Social influences	6	2	9
Behavioural regulation	10	6	10
Attitude towards the behaviour^b^	10	5	10
Self‐image	2	2	4
Feedback processes	9	6	10
Social learning and imitation	5	2	
Behavioural cueing	7	5	10

^a^ This MoA incorporates both motivation *and* intentions.

^b^ This MoA incorporates both attitude towards the behaviour *and* general attitudes and beliefs.

**FIGURE 1:**
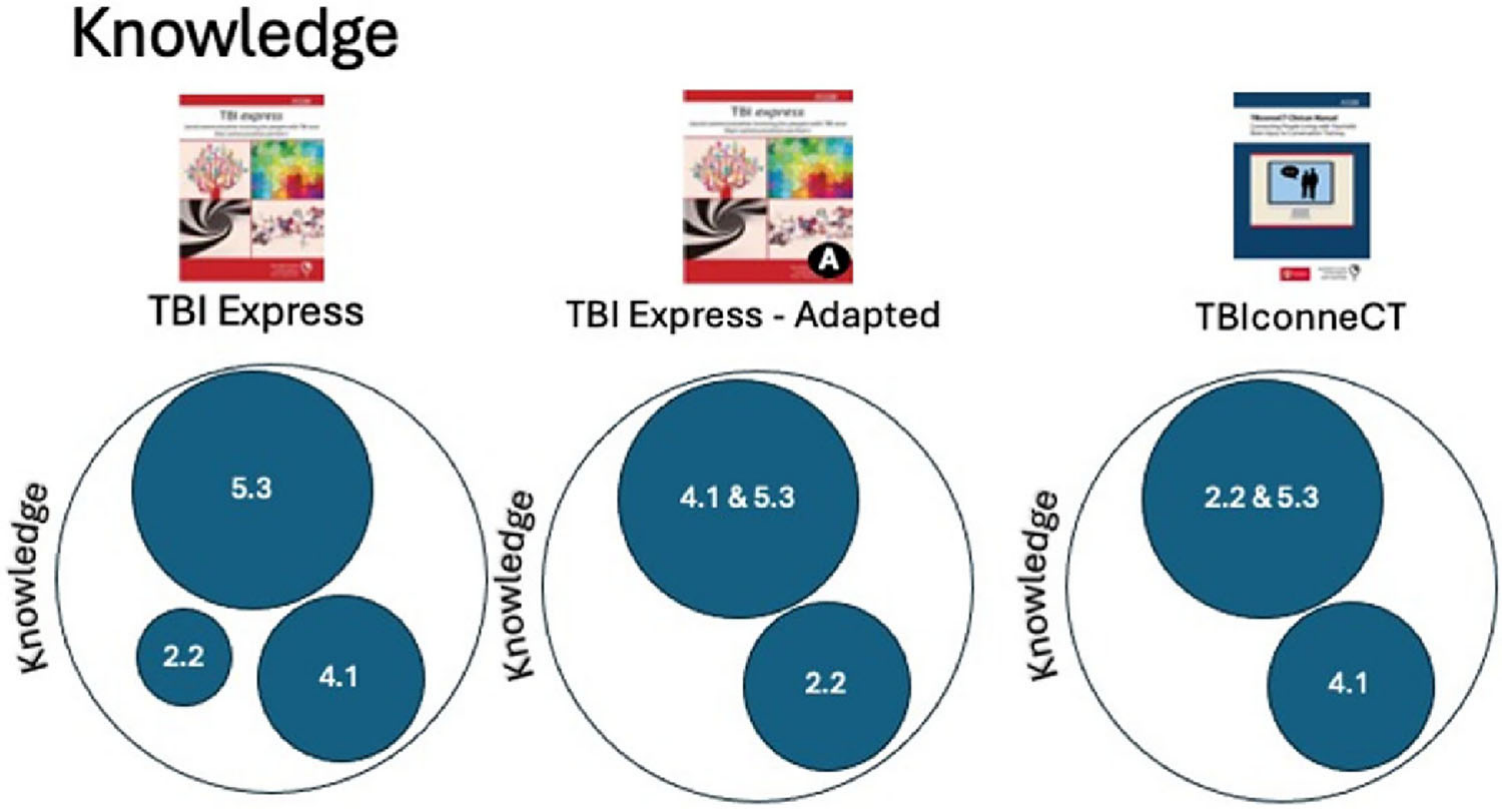
Example of the Knowledge MoA, and the BCTs present for each programme, expressed as the % degree (large circle = 80–100% of modules; medium circle = 30–80% of modules; small circle = <30% of modules) with which those BCTs are present within a session module of a programme.

To further understand the information about the BCTs and MoAs, this information was then combined to understand the relative presence of BCTs for each individual MoA (see an example of the knowledge MoA, Figure 1). The degree with which a BCT was present for a MoA is expressed as a percentage of the number of session modules in which it was present for that programme. This was either 80–100% of modules (large circle), 30–80% of modules (medium circle), or <30% of modules (small circle). Figure 1 provides an example of this for the *Knowledge* MoA. For TBI Express, the BCT *information about social and environmental consequences* (5.3) was present in >80% of session modules, *instruction on how to perform the behaviour* (4.1) was present in 30–80% of session modules and *feedback on behaviour* (2.2) present in <30% of session modules. For TBI Express‐Adapted, the BCTs *information about social and environmental consequences* (5.3) and *instruction on how to perform the behaviour* (4.1) were present in >80% of session modules, and *feedback on behaviour* (2.2) present in 30–80% of session modules. In TBIconneCT, *information about social and environmental consequences* (5.3) and *feedback on behaviour* (2.2) was present in >80% of session modules and *instruction on how to perform the behaviour* (4.1) was present in 30–80% of session modules. In other words, all three programmes value the BCTs 5.3, 4.1 and 2.2 as present in the *knowledge* MoA, but to varying degrees. The remaining 15 MoAs are shown in a similar form in Supplementary Material B.

## 4 Discussion

This study examined CPT interventions for adults with ABI by applying behaviour change theory to published intervention manuals to identify their active components. Coding of three evidence‐based CPT programmes identified a wide range of BCTs and MoAs, providing support for the complexity of CPT interventions, as delivered by SLTs to individuals with cognitive‐communication impairments and their CPs. Across the three programmes, between 20–25 BCTs linked to 15–16 MoAs were identified, representing approximately one‐third of all possible BCTs and nearly two‐thirds of all possible MoAs.

Although CPT interventions are commonly assumed to target conversation as the primary behaviour, this study highlights challenges in specifying conversation as a discrete target behaviour, given its breadth and the multiple behaviours involved (e.g., ask questions, take turns).

Further examination of intervention content revealed 27 distinct BCTs across the three CPT programmes. The use of behaviour change theory to identify active ingredients is not uncommon (Howlett et al. 2019), and coding BCTs from intervention manuals has been shown to identify a greater number of BCTs than when coding intervention descriptions in published studies (Lorencatto et al. 2013). However, relatively few studies have identified BCTs solely from intervention manuals (Johnson et al. 2021; Lorencatto et al. 2013). A previous study applied the intervention taxonomy (Schulz et al. 2010), to describe key intervention features from the TBI Express manual (O'Rourke et al. 2018). The taxonomy is a classification system that describes interventions according to delivery (e.g., mode, materials and location) and content and goals of interventions (treatment content strategies, mechanisms of action). O'Rourke et al. (2018) identified 11 different skill‐building techniques including the use of role‐play, demonstration, facilitate discussion and practice conversation. While there is overlap of techniques in each of these studies, the comprehensive and systematic approach afforded by behaviour change theory yielded a far greater number of BCTs across the three programmes coded. This finding would suggest BCTs bring a higher level of specificity as some of the previous skill‐building techniques such as ‘*facilitate discussion*’ may relate to multiple BCTs (e.g., problem solving, goal setting and giving feedback). The higher number of BCTs may also provide evidence to highlight the complex nature of CPT that is needed to be delivered by skilled clinician.

The coding of the three CPT programmes allowed for the comparison of BCTs between each of them. From the 27 BCTs identified, 19 were similar across all three programmes including frequently coded BCTs such as *goal setting (behaviour) (1.1), behavioural contract (1.8), feedback on behaviours (2.2), information about social and environmental consequences (5.3) and behavioural practice/rehearsal (8.1)*. These findings highlight the importance of setting goals, providing feedback on goals, information about the consequences of impaired communication and opportunities for practice (both within and outside of treatment sessions). Of the 27 BCTs, 16 were identical to those identified from a programme intended for people with post‐stroke aphasia (Johnson et al. 2021).

This study is the first to have systematically linked the relevant BCTs to the hypothesised MoAs, within CPT, enabling a more detailed exploration of the processes through which change in communication behaviour may occur. Across the three programmes, all treatment sessions targeted multiple MoAs (*Knowledge, Skills, Beliefs about capabilities, Goals* and *Behavioural regulation*). This pattern suggests that to influence changes in communicative behaviour, CPT needs to increase knowledge and change attitudes, build confidence, increase motivation, improve skills and increase independence. Similarly, Johnson et al. (2017) identified multiple MoAs related to changes in awareness, attitude, priorities and skills in CPT for post‐stroke aphasia, highlighting that multiple mechanisms are needed to influence behaviour change. Together, these findings support the complexity of CPT, in which behaviour change occurs from multiple interacting MoA, where multiple BCTs influence a single MoA. This approach enables interventions to be deliberately and precisely designed around the mechanisms most likely to influence change, with BCTs selected to directly target those mechanisms, thus providing a strong theoretical basis for developing behavioural interventions (Connell et al. 2019).

The findings of this study highlight the skills required by the SLT to deliver multiple BCTs to bring about change in a range of MoAs. There were some BCTs that did not emerge despite a conclusive link to a specific MoA, such as highlighting the *discrepancy between current behaviour and goal* or *review outcome goal* for the goal MoA. For example, *review outcome goal* might involve an explicit review of outcomes such as more positive conversations, improved relationships or fewer conflicts. This finding suggests that either the BCTs had not previously been considered and should be in the future, or there was insufficient detail in the manual to warrant coding of a specific BCT. However, there may be some BCTs that would not be appropriate for a CPT intervention (e.g., *biofeedback*). The BCT‐MoA links are based on changing people's behaviours from health risks including, smoking, alcohol misuse and diabetes (Michie et al. 2021) so some BCTs, for example, the use of biofeedback to change knowledge may not be required in a CPT intervention.

The presence of different BCTs and MoAs does not demonstrate which BCTs are most effective in changing a MoA. In fact, it is unclear whether a single BCT or a package of BCTs is required for making a change in behaviour. Johnson et al. (2021) suggested that to reduce barrier behaviours, a combination of three BCTs may help to support change. For example, involving the provision of information about the social and emotional reasons to not use a particular behaviour and to use an alternative in its place. Adoption of new behaviours was considered to potentially be more complex, involving multiple MoAs and a package of BCTs. Meta‐regression has previously been used to determine the effects of individual BCTs for physical activity interventions (Howlett et al. 2019). In that study, four BCTs were found to have larger effect sizes at post‐treatment and six at follow‐up however, the BCTs were not linked to MoAs and the findings could not determine whether it was a single BCT or package of BCTs that was required to influence behaviour change. As some MoAs were targeted within multiple sessions (>80% of the time), the finding may suggest that targeting the same MoA frequently, with the same package of BCTs, may be required for enacting behaviour change.

The person to whom the BCT is directed was not clear from this study (i.e. the person with ABI *or* the CP). This is important as some techniques may be better received and understood by one person compared to another. For example, a person with ABI may require different techniques due to their cognitive difficulties. Previous qualitative studies have reported potentially positive and effective techniques such as the use of role‐play, demonstration and practice (Avramović et al. 2023; Behn et al. 2015; Togher et al. 2012b). However, many of these reports were made by CPs involved in the training suggesting that these techniques are most helpful to them. Being clear about what the BCT is *and* who it is directed to will be important for future delivery of CPT.

There was one programme, TBIconneCT which emerged with more BCTs compared to the other two programmes. In that programme, four additional BCTs were identified, which were not found in the other two programmes. These included: *monitoring of emotional consequences (5.4)*; *social reward (10.4)*; *adding objects to the environment (12.5)*; and *valued self‐identity (13.4)*. While these may reflect changes to the content of the programmes, they seem more likely related to the lack of information in the earlier programmes to code those BCTs. For example, ‘*social reward*’ refers to positive reinforcement and statements of praise and encouragement. While this was not explicitly mentioned in the earlier programmes, it may be assumed that a clinician should provide social reward as part of the rehabilitation programme. The TBIconneCT programme was published a decade after TBI Express and was likely to have been influenced by intervention description and development frameworks, including the rehabilitation treatment specification system (RTSS: Meulenbroek et al. 2019) and the template for intervention description and replication (TIDieR: Hoffmann et al. 2014). Such frameworks encourage researchers to provide more detailed and comprehensive manuals to enable translation into practice (Lorencatto et al. 2013).

One aspect of the training that was not clearly defined was the target behaviour. Identification of a target behaviour is central to behaviour change theory and other rehabilitation intervention frameworks, such as the RTSS also highlight the importance of having a clear target for rehabilitation. The manuals in this study did not clearly specify the target behaviour nor was it clear whether the behaviour was focused on the person with ABI or the CP. In the current study, the target behaviour was agreed to be conversation; however, this target may be perceived as too broad as many behaviours (e.g. ask questions, take turns, give time to respond) can contribute to the relative success (or not) of a conversation. Changes to conversation may be more akin to an outcome alongside communicative participation, perceived communicative ability or increased social activities (Behn et al. 2025). Given that conversation is a combination of discourse genres including, for example, storytelling, social chat, and observations, comments and questions (Eggins and Slade 1997), it is not surprising that it is difficult to classify it as a target behaviour. Therefore, for any training to be undertaken, clinicians need to first define the target behaviour (or strategy to use) for *both* the person with ABI and CP.

Reliability for coding was moderate to almost perfect for two of the manuals. Use of the first programme, TBI Express to pilot code and develop a set of coding guidelines is likely to have improved the reliability process, as has been done previously (Presseau et al. 2015). The kappa coefficients were stronger than for other studies (Bird et al. 2013; Crawshaw et al. 2022; Johnson et al. 2021) and percent agreement was, consistent with other studies that coded manuals (Johnson et al. 2021; Lorencatto et al. 2013). Despite these findings, there were challenges with coding. Some activities were underspecified and matching a BCT to a MoA was not always clear from the activity described. There is a degree of subjectivity which may affect the judgement of the presence of BCT based on how closely the definition approximates the description in the programme. Using raters with training in BCT coding, clinical experience of the intervention under investigation and engaging in frequent coding discussions ensured the programmes were coded as reliably as possible. However, challenges with coding BCTs highlight the need for clear guidelines to be agreed at the outset of the rating process including, further clarification and refinement to address a nuanced meaning of BCTs and MoAs for this clinical population (Abraham et al. 2015; Johnson et al. 2021; Presseau et al. 2015). Presently, the existing definitions of each of the BCTs and MoAs and examples are more suited to changing health behaviours rather than changes from a rehabilitation healthcare intervention. Re‐framing of the BCTs using simplified accessible language with specific examples framed for CPT in brain injury may be useful and as such is under development for a future study by the research team.

One of the limitations of this study is that only two modules from each manualised programme (representing 20–33% of all content) were coded for reliability and coding of the entire manual *could* have been considered. Despite this, having a pair of raters with experience in behaviour change theory, ABI and CPT ensured that agreement for identifying BCTs was no more than 10% different to that achieved by Lorencatto et al. (2013). Only one theory or framework was used for the identification of BCTs and MoAs in this study. Behaviour change theory was not initially intended for describing interventions delivered to individuals with cognitive impairment. Since the completion of this research, the behaviour change intervention ontology (BCIO) has emerged and may be used for coding (Marques et al. 2024) although 281 BCTs are proposed which may present an additional challenge for reliable coding. Other intervention description frameworks such as the RTSS have been proposed for the description of interventions and have agreed by expert consensus thematic categories of techniques for social communication interventions for people with ABI (e.g., repeated practice, feedback, strategy instruction, group process activities) (Meulenbroek et al. 2019). However, a far greater number of BCTs and MoAs were identified using behaviour change theory which may more clearly illustrate the complexity of the intervention. There are often areas of commonality between theories and frameworks of intervention description and development and future steps may be to draw on the strengths of each to develop an intervention in the future.

## 5 Conclusions

Applying behaviour change theory to TBI express and its variants revealed unique insight into the complexity and multicomponential nature of CPT. This research has identified a core list of potentially active ingredients (mechanisms and techniques) required to deliver CPT to individuals with ABI and their CPs. Partners are anticipated to improve their communication behaviours via capabilities, skills and regulation, through SLT‐delivered CPT which focuses most commonly on goal setting, feedback, information about consequences and practice. Such insights are gathered to further refine and adapt existing interventions and develop new ones. The challenge of distinguishing between target behaviours and desired outcomes highlights the need for further refinement of techniques and conceptual work for SLTs to distinguish the delivery of techniques. Future work should aim to identify the mechanisms and techniques considered most important to practicing clinicians to design and test the feasibility of an adapted CPT programme for ABI in public healthcare services. By underpinning the intervention theoretically and engaging those involved in the delivery and receipt of CPT in ABI, more clearly described interventions can be developed to ensure better replication and translation into clinical practice. Thus, ensuring that individuals with ABI and their CPs get access to well‐evidenced interventions aimed to improve their communication and relationships with others.

## Funding

Nicholas Behn is supported by an NIHR Advanced Fellowship [NIHR302952]. This research was funded by the NIHR. The views expressed in this publication are those of the author(s) and not necessarily those of the NIHR or the Department of Health and Social Care.

## Conflicts of Interest

The authors declare no conflicts of interest.

## Supporting information




**Supplementary Material A**. Coding discrepancies. **Supplementary Material B**. List of each mechanism, and the BCTs present in each programme, expressed as the % degree with which those BCTs are present within a module of a programme, either 80–100% of modules, 30–80% of modules, or <30% of modules. Blank circles indicate no BCTs present for that mechanism.

## 1

**TABLE A:** Complete list of the session modules for each of the three coded programmes.

Name	TBI Express	TBI Express‐Adapted	TBIconneCT
Module 1	Introductions	Introductions	Introductions
Module 2	TBI and Communication	TBI and Communication	TBI and Communication
Module 3	Conversation Formulas	Collaboration	Playing Your Role
Module 4	Conversation Formulas	Elaboration	Effective Speaking and Listening
Module 5	Collaboration	Asking Questions	Collaboration—Working Together
Module 6	Extending Conversations	Putting it all Together	Elaboration—Extending
Module 7	Better Questions	—	Asking Questions
Module 8	Improving Skills and Confidence	—	Putting it all Together 1
Module 9	Improving Skills and Confidence	—	Putting it all Together 2
Module 10	Planning for the Future and Group Celebration	—	Putting it all Together 3
Length of manual	226 pages^a^	178 pages	245 pages

^a^ The TBI Express manual is 409 pages in length. Only sections 5, 1, 2.5 were coded as these sections are relevant to the training of communication partners; remaining sections are focused on training the person with brain injury only and not the communication partners.
