# Supplementary material for: Behaviour Change Techniques and Mechanisms of Action: Identification of the Active Ingredients in Communication Partner Training for People With Acquired Brain Injury
Source: Int J Lang Commun Disord. 2026 Apr 13;61:e70238. doi: 10.1111/1460-6984.70238 (PMC13071541; doi:10.1111/1460-6984.70238)

Supplementary Material A. Coding discrepancies*

| Module/BCT | Activity description | Nature of disagreement | Decision |
| --- | --- | --- | --- |
| TBI-Adapted (Module 2) | | | |
| 3.1 Social support (unspecified) | Group discussion about a role-played conversation | Not coded by one rater | Do not include as no reference to target behaviour or outcome |
| 5.6 Information about emotional consequences | A single sentence in handout (H2-4) as to how awkward situations make people feel bad about a situation | Not coded by one rater | Do not include as not a clear activity in the session plan. Reference was made in handout. |
| 6.3 Information about others approval | Playing a home practice video for the group which is discussed | Coded by both raters as 2.2. One rater additionally used 6.3 code. | Do not include as insufficient detail in activity to code (use 2.2 only) |
| TBI-Adapted (Module 5) | |  |  |
| 1.3 Goal setting (outcome) | Identification of weaknesses and areas to work on. | Coded by both raters as 1.1. One rater additionally used 1.3 code. | Include (also code as 1.1) |
| 2.4 Self-monitoring of outcome(s) of behaviour | Get feedback from dyads on the effects of different question types | Coded by second rater as 2.2 | Include (also code as 2.2) |
| 3.1 Social support (unspecified) | Group discussion about a role-played conversation | Not coded by one rater | Do not include as no reference to target behaviour or outcome |
| 13.2 Framing/reframing | Practicing the different question types (open/closed and simple/complex) and consider the difference between them to changing perspective on question types | Coded by both raters as 2.2, 2.7 and 5.3. One rater additionally used 13.2 code. | Include (also code as 2.2, 2.7 and 5.3) |
| TBIconneCT (Module 3) | |  |  |
| 2.2 Feedback on behaviour | Provide feedback on strategy effectiveness and the positives from a discussion of a home practice recording and conversation practice video | Coded by second rater as 2.7 | Include (also code as 2.7) |
| 8.6 Generalisation of target behaviour | Identifying communication topics and communication challenges to complete before next session | Coded by both raters as 1.1. One rater additionally used 8.6 code. | Include (also code as 1.1) |
| 8.7 Graded tasks | Identifying communication challenges to work on between sessions | Not coded by one rater | Do not include as insufficient detail in activity to code |
| 13.2 Framing/reframing | Discussion about how conversation is like acting and how good communication involves playing a role successfully. | Not coded by one rater | Include |
| 15.4 Self-talk | Coded from a handout where there was an example reference to giving yourself a pep talk for positive communication | Not coded by one rater | Do not include as not a clear activity in the session plan. Reference was made in handout. |
| TBIconneCT (Module 10) | |  |  |
| 1.7 Review outcome(s) goal | Rating the achievement of each goal set during the programme | Coded by second rater as 1.5. | Do not include (code conservatively as 1.5). |
| 3.1 Social support (unspecified) | Identification of what will help continue progress and how regular check-ins may help | Not coded by one rater | Include |
| 3.2 Social support (practical) | Identification of what will help continue progress and how asking for prompts and feedback from others may help | Coded by second rater as 3.1 | Do not include (code conservatively as 3.1) |
| 4.1 Instruction on how to perform the behaviour | Use of a toolkit of communication strategies to help work out what strategies work best and are most relevant for the dyad | Not coded by one rater | Include |

*Refer to the BCTTv1 for labels and descriptions of behavioural change techniques (https://digitalwellbeing.org/wp-content/uploads/2016/11/BCTTv1_PDF_version.pdf)

Supplementary Material B. List of each mechanism, and the BCTs present in each programme, expressed as the % degree with which those BCTs are present within a module of a programme, either 80-100% of modules, 30-80% of modules, or <30% of modules. Blank circles indicate no BCTs present for that mechanism.


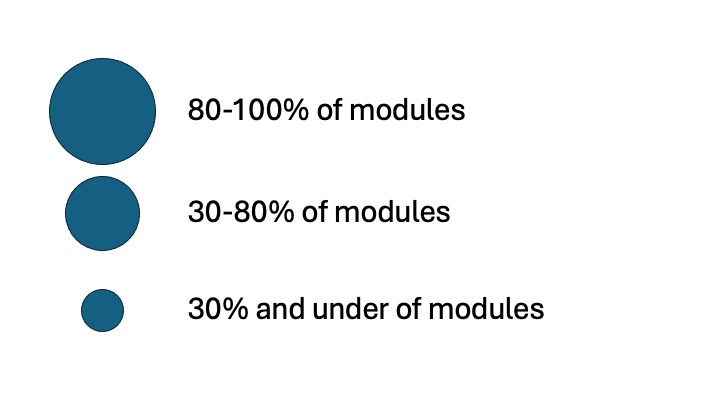


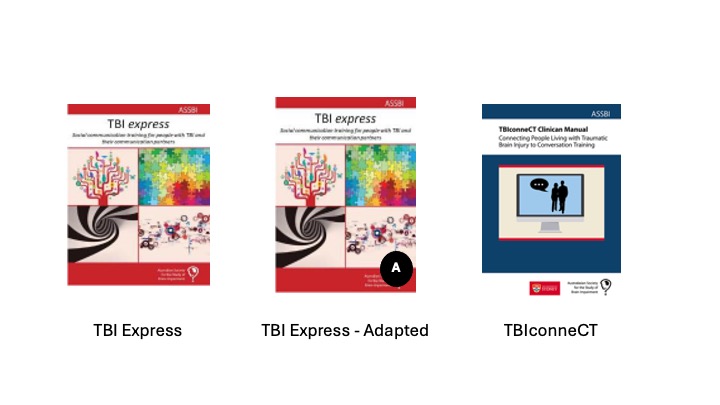


**List of identified BCTs**

| Behavioural Change Technique (BCT) | |
| --- | --- |
| 1.1 | Goal setting (behaviour) |
| 1.2 | Problem Solving |
| 1.3 | Goal setting (outcome) |
| 1.4 | Action planning |
| 1.5 | Review behaviour goal(s) |
| 1.8 | Behavioural contract |
| 2.2 | Feedback on behaviour |
| 2.3 | Self-monitoring of behaviour |
| 2.4 | Self-monitoring of outcome(s) of behaviour |
| 2.7 | Feedback on outcome(s) of behaviour |
| 3.1 | Social support (unspecified) |
| 3.2 | Social support (practical) |
| 4.1 | Instruction on how to perform the behaviour |
| 5.3 | Information about social and environmental consequences |
| 5.4 | Monitoring of emotional consequences |
| 6.1 | Demonstration of the behaviour |
| 7.1 | Prompts/cues |
| 8.1 | Behavioural practice/rehearsal |
| 8.2 | Behaviour substitution |
| 8.3 | Habit formation |
| 8.6 | Generalisation of target behaviour |
| 8.7 | Graded tasks |
| 10.4 | Social reward |
| 12.5 | Adding objects to the environment |
| 13.2 | Framing/reframing |
| 13.4 | Valued self-identity |
| 15.3 | Focus on past success |


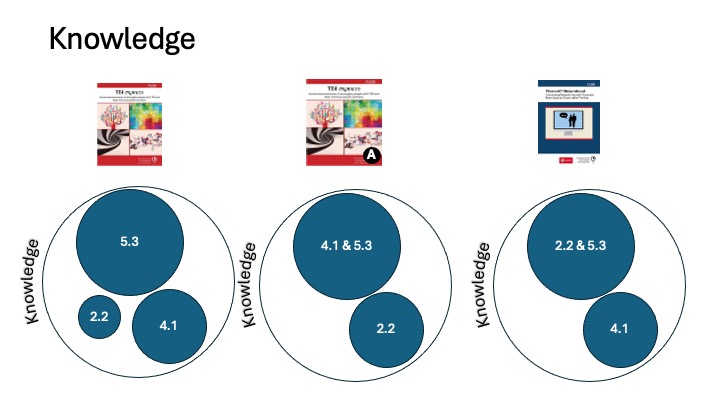


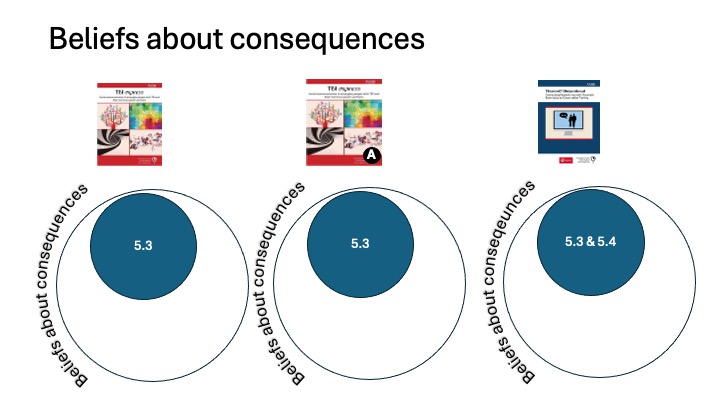


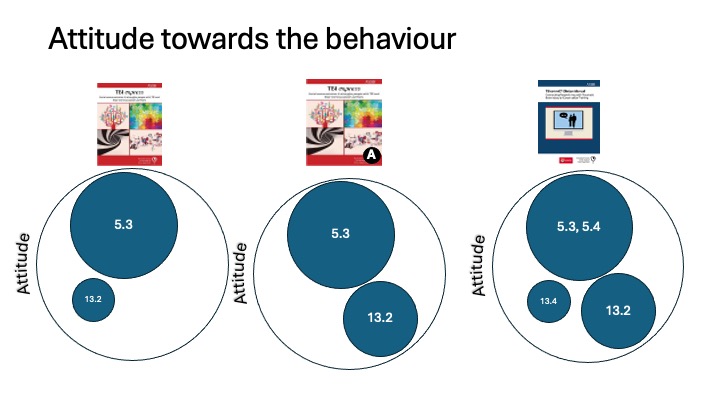


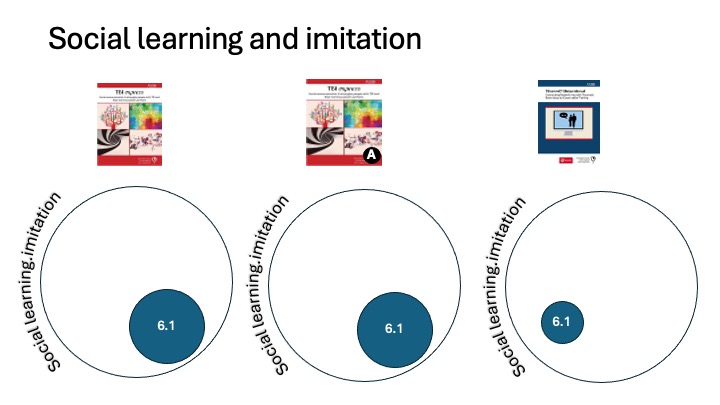


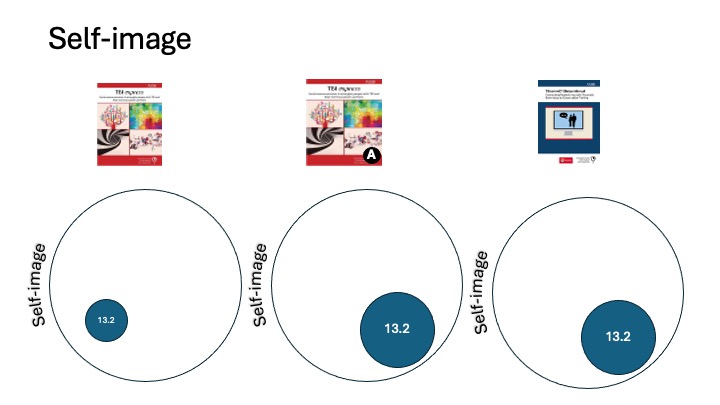


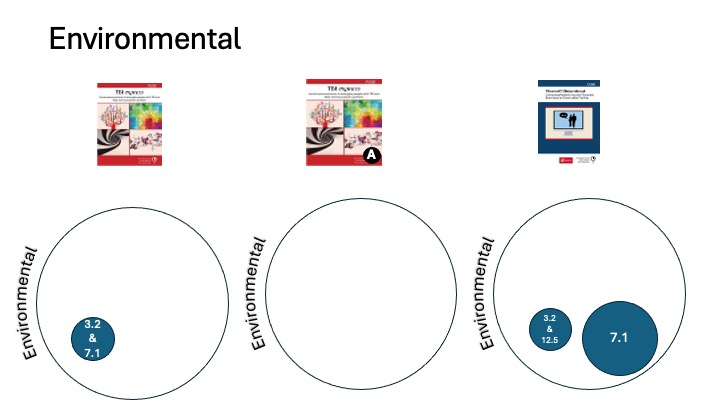


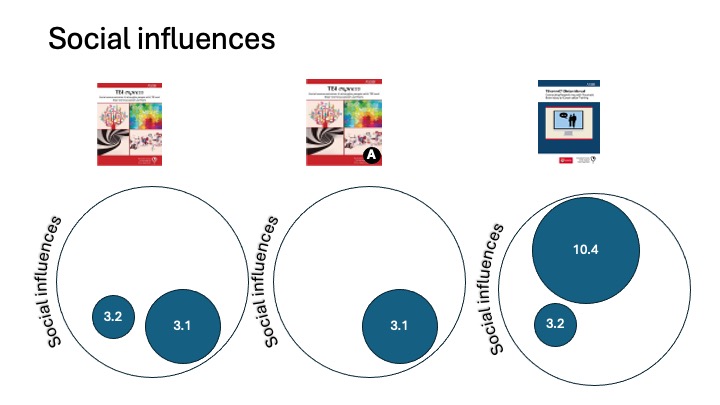


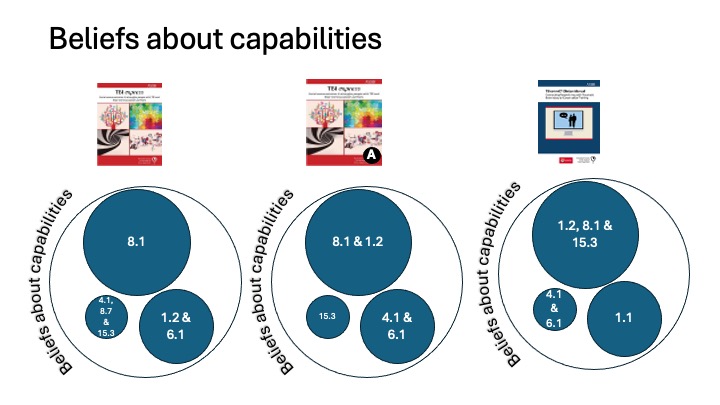


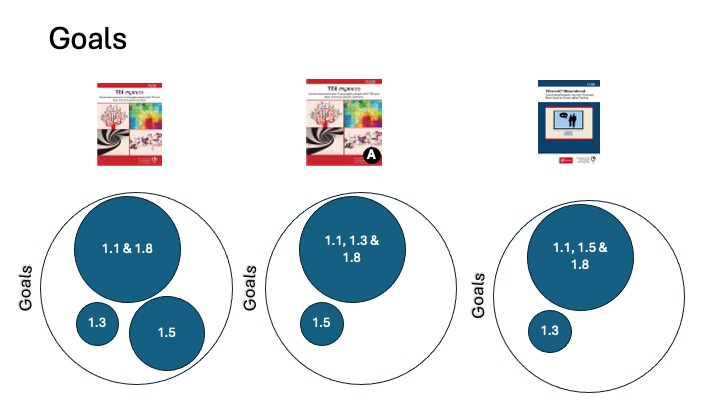


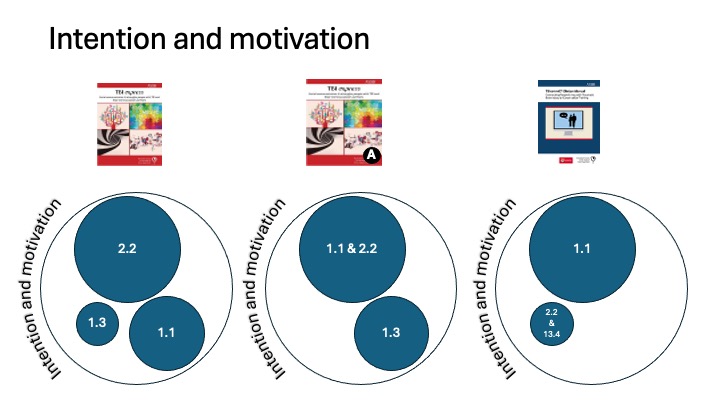


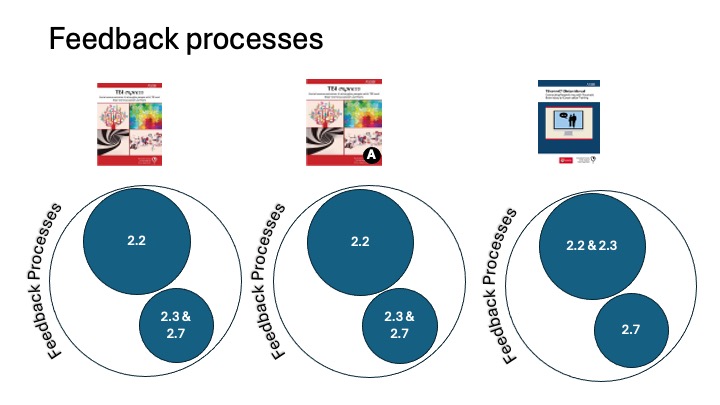


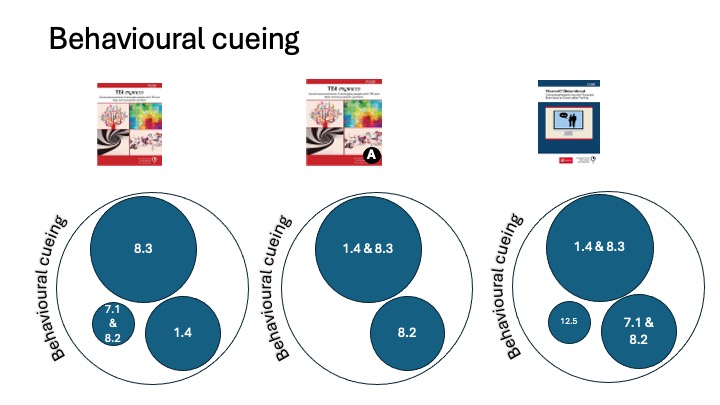


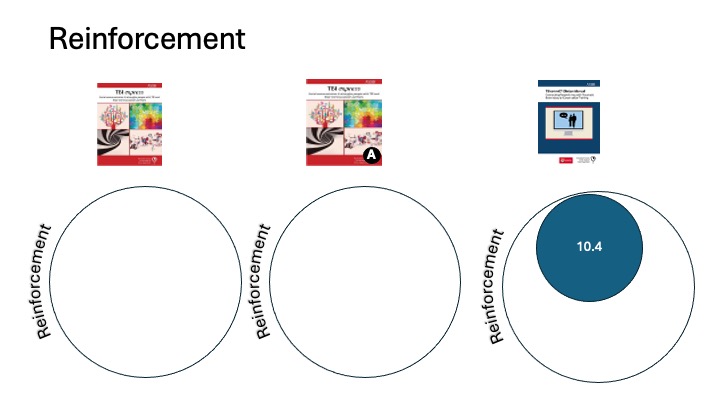


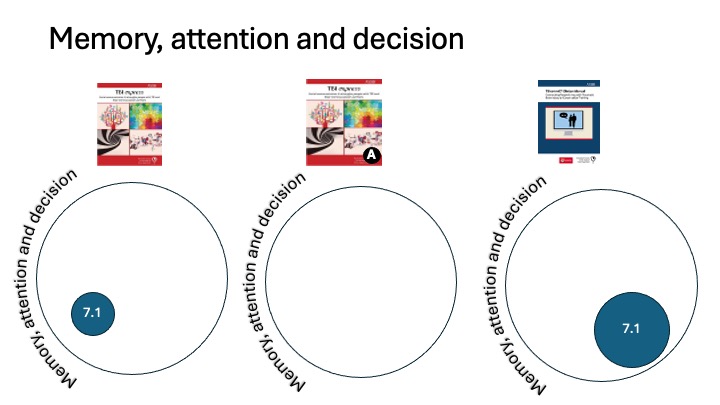


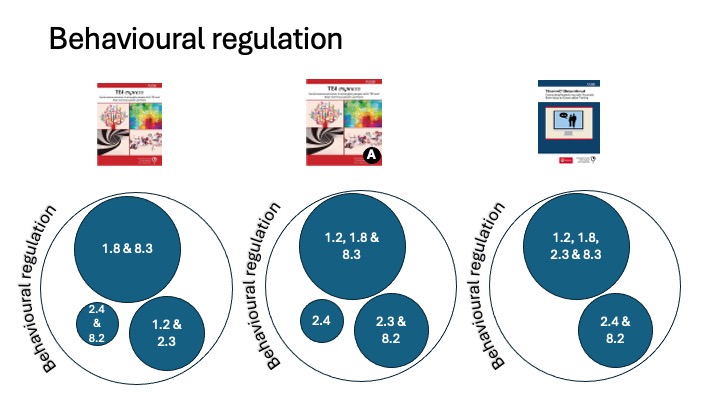

Supplement: Supplementary file 1 — Supplementary Material A. Coding discrepancies. Supplementary Material B. List of each mechanism, and the BCTs present in each programme, expressed as the % degree with which those BCTs are present within a module of a programme, either 80–100% of modules, 30–80% of modules, or <30% of modules. Blank circles indicate no BCTs present for that mechanism. [file JLCD-61-0-s001.docx]
